# Burning of the Quarantine Buildings at Staten Island

**Published:** 1858-10

**Authors:** 


*Burning of the Quarantine Buildings at Staten Island.*—We read with the most lively indignation and horror the accounts which appeared in the New York papers of this savage outrage, and had intended to express our feelings on the subject in the pages of the Journal. We forego this, however, and copy an article from the New York Journal of Medicine, the editor of which of course has better opportunities of judging of the prevalent feeling in regard to the matter.

YELLOW FEVER—QURANTINE: *Destruction of the Quarantine Buildings by a Mob.*—The New York Quarantine—its uses, abuses, and the ruthless burning of all hospital and other buildings pertaining thereto, have suddenly become the exciting topics of the day. As this number of the Journal is going to press, the last combustible structure at Quarantine is being burned to the ground.

By these barbarous acts of incendiarism it is possible that the citizens of this metropolis, and the people of the State of New York, may be aroused to some proper sense of the importance and the practical bearings of the various and vexed questions relating to our Quarantine system. "It is an ill wind that blows no good," and if this daring act of the people of Staten Island could be the means of rectifying the abuses of Quarantine at this great entrepôt of American commerce, and could such riotous proceedings become the means of procuring the establishment of a rational and efficient *sanitary system* for the port and city of New York, this terrible experience were cheaply earned, though it is likely to cost the State nearly a half million dollars.

We have not time for comments on this unparalleled and inhuman firing of hospitals. With pain we have looked upon the strange scene of fever patients dragged forth from their comfortable wards and exposed without shelter in the open air, while the edifices which were erected for their benefit, and for the protection of community from infectious maladies, were smouldering in ruins from the torch of American citizens. With shame for the medical profession, and humiliation for the boasted intelligence and social and civil superiority of the Empire State, are we compelled to acknowledge that these high-handed, unrestrained and unparalleled outrages against the laws and the dignity of the State are the direct result of the shameless prostitution of the most important responsibilities, executive and legislative. to the basest purposes, while Legislators, Governors, and Boards of Health have found ample apology, and sheltered themselves behind the ignorance and dissensions of medical writers on infectious diseases and quarantine.

Difficult as it may be to determine certain questions relating to the sanitary protection of a great commercial city like New York, the time has come when our Quarantine regulations must be established upon some clearly-defined and rational basis which will command the respect of the people, and *actually* guard the public from imported infection.

The events affecting the question of the imported origin of yellow fever in the port of New York, and in the vicinity of the Quarantine station, this season, as in past years, have been peculiarly definite and instructive. The cases in New York and Brooklyn, thus far, have occurred only in persons employed on the cargoes of vessels from infected ports, or in the work of cleansing and repairing such vessels.

On the eastern shore of Staten Island the infection of the fever is believed to have become localized at two or three distinct and widely-separated points. Some twenty cases have occurred, and about one-third the number have died with black vomit.

The number of sick seamen admitted to the hospital from infected vessels has been usually large, and several stevedores, shipkeepers, and other employees on such vessels in Quarantine, have been brought into the fever wards. The U. S. steamer "Susquehanna," which arrived with yellow fever from the Gulf of Mexico, early in the season, has visited death upon a large proportion of all unacclimated persons who have visited that vessel since her arrival; and other vessels, without cargo, have proved to be similarly infected.

No other port in the world presents equal facilities for the successful study of those laws that govern the great scourge of the American topics as a transportable infection. Here at the periphery of that variable zone, within which the infection of yellow fever may be reproduced and yield its fatal fruits, all the facts relating to introduction and diffusion among us are replete with interest, and from them may eventually be evolved the laws which govern the etiology of the disease that has baffled the closest study from the days of Chisholm, Rush, Pym, and Bayley.

*Obituary Notices.*—We glean from our exchanges and the public prints, the following obituary notices of medical men:

A meeting of the Medical Society of the county of Erie, was held at the rooms of the Buffalo Medical Association on Saturday afternoon, the 18th Sept., to pay the respect due to the momory and services of Dr. H. H. Bissell.

The society was called to order at 4 o'clock, by the President, Prof. Flint, who stated the object of the meeting.

After appropriate remarks, on motion of Dr. Strong, a committee was appointed to draft resolutions appropriate to the occasion. Drs. Strong, Wyckoff and Gay were appointed such committee, and reported the following resolutions, which were unanimously adopted:

Whereas, by an afflictive Providence, Dr. H. H. Bissell, one of our senior practitioners, and for more than a quarter of a century a member of this society, has been suddenly stricken down and removed from his earthly labors and associations by death, as an expression of the sentiments of this society,

*Resolved*, That by this event our profession loses and mourns one of its most extensive, laborious and widely respected practitioners.

*Resolved*, That we hereby respectfully tender to the stricken widow and family an expression of our condolence and sympathy in this hour of bereavement and sorrow. In further token of which,

*Resolved*, That we will attend the funeral of our deceased associate and fellow member in a body.

*Resolved*, That a copy of our action herein be transmitted by the secretary to the bereaved widow, and one likewise be furnished for publication.

The committee on resolutions were directed to select from among the members of the society, the usual number of pall bearers.

The society then adjourned.

Dr. Robert Hare, Professor of Chemistry in the University of Pennsylvania for nearly thirty years; discoverer of the oxy-hydrogen blow-pipe; author of "Compendium of Chemistry," written as a text-book for his students, died on the 16th of May last, at the advanced age of 77 years.

*Shelby Medical College.*—We have at last received the announcement of the Shelby Medical College, Nashville, Tennessee. This new institution goes into operation in the fall, and with the following gentlemen composing the faculty, viz: Dr. John F. May, Professor of Surgery; Dr. E. B. Haskins, Professor of Theory and Practice; Dr. John P. Ford, Professor of Obstetrics, etc.; Dr. S. L. Maddin, Professor of Anatomy; Dr. John H. Callender, Professor of Materia Medica; Dr. R. O. Curry, Professor of Chemistry, etc.; Dr. D. F. Wright, Professor of Physiolgy, etc.; Dr. H. M. Compton, Demonstrator of Anatomy. The faculty announce that they will be thoroughly prepared to teach medicine properly, and we wish them the fullest success in their enterprise.—*New Orleans Med. News and Hospital Gazette.*

---

*Professorial Change.*—Dr. Daniel F. Wright, of Memphis, Tenn., desires to announce his resignation of the chair of Physiology and Pathology in the Memphis Medical College, and his acceptance of the same chair in the Shelby Medical College, at Nashville. Dr. Wright was editor of the Memphis Medical Recorder, and now that he becomes a colleague of Dr. Richard O. Curry, the respected editor of the Southern Journal of the Medical and Physical Sciences, we should not be surprised soon to find on our table a fusion journal. Two such editors would create a stir amongst rivals.—*Ibid.*

---

*Thayer's Fluid Extract.*—These preparations have now become almost indispensable to the practitioner, from their elegance and reliability, and Mr. Henry Thayer, whose advertisement appears in this number, is now acknowledged to be surpassed by none in their manufacture. Mr. Thayer was kind enough to give us an opportunity of testing his extracts practically, and speaking from actual experience, we can say that they are as reliable as could be desired. The fluid extract of belladonna was used for a time by Prof. Flint at the Buffalo Hospital of the Sisters of Charity, with excellent effect, and we now have a patient under our charge who is taking the fluid extract of Gentian with the same results. In the latter case we omitted the remedy for a day, but were compelled to come back to it immediately. We are glad to add our testimony to the excellence of Thayer's Fluid Extracts, which we can do from our own experience.

*Tilden's Pharmaceutic Sugar Coated Pills and Granules.*—We have received from the manufacturers, by the politeness of Mr. A. I. Mathews, specimens of these preparations. If we can ever be able to obtain disagreeable remedies disguised in this way, we will have made a great point against our milk and sugar enemy, the homœopathic quack; Mr. Tilden is giving us valuable aid in this respect by means of his sugar-coated pills. We have made trial of some of them, and find them quite as effectual as the remedy in any other form, without any disagreeable taste. For sale by A. I. Mathews, No. 220 Main street, Buffalo.

